# Coinfection of tick cell lines has variable effects on replication of intracellular bacterial and viral pathogens

**DOI:** 10.1016/j.ttbdis.2014.01.010

**Published:** 2014-06

**Authors:** Anna Moniuszko, Claudia Rückert, M. Pilar Alberdi, Gerald Barry, Brian Stevenson, John K. Fazakerley, Alain Kohl, Lesley Bell-Sakyi

**Affiliations:** a The Roslin Institute and Royal (Dick) School of Veterinary Studies, University of Edinburgh, Easter Bush, Midlothian EH25 9RG, Scotland, UK; b Department of Infectious Diseases and Neuroinfections, Medical University in Białystok, Żurawia 14, 15-540 Białystok, Poland; c Department of Microbiology, Immunology and Molecular Genetics, University of Kentucky College of Medicine, MS421 Chandler Medical Center, 800 Rose Street, Lexington, KY 40536-0298, USA; d The Pirbright Institute, Ash Road, Pirbright, Woking, Surrey GU24 0NF, UK

**Keywords:** Tick cell line, Coinfection, *Ehrlichia*, *Borrelia*, Semliki Forest virus, *Ixodes* spp.

## Abstract

Ticks transmit various human and animal microbial pathogens and may harbour more than one pathogen simultaneously. Both viruses and bacteria can trigger, and may subsequently suppress, vertebrate host and arthropod vector anti-microbial responses. Microbial coinfection of ticks could lead to an advantage or disadvantage for one or more of the microorganisms. In this preliminary study, cell lines derived from the ticks *Ixodes scapularis* and *Ixodes ricinus* were infected sequentially with 2 arthropod-borne pathogens, *Borrelia burgdorferi* s.s., *Ehrlichia ruminantium*, or Semliki Forest virus (SFV), and the effect of coinfection on the replication of these pathogens was measured. Prior infection of tick cell cultures with the spirochaete *B. burgdorferi* enhanced subsequent replication of the rickettsial pathogen *E. ruminantium* whereas addition of spirochaetes to cells infected with *E. ruminantium* had no effect on growth of the latter. Both prior and subsequent presence of *B. burgdorferi* also had a positive effect on SFV replication. Presence of *E. ruminantium* or SFV had no measurable effect on *B. burgdorferi* growth. In tick cells infected first with *E. ruminantium* and then with SFV, virus replication was significantly higher across all time points measured (24, 48, 72 h post infection), while presence of the virus had no detectable effect on bacterial growth. When cells were infected first with SFV and then with *E. ruminantium*, there was no effect on replication of either pathogen. The results of this preliminary study indicate that interplay does occur between different pathogens during infection of tick cells. Further study is needed to determine if this results from direct pathogen–pathogen interaction or from effects on host cell defences, and to determine if these observations also apply in vivo in ticks. If presence of one pathogen in the tick vector results in increased replication of another, this could have implications for disease transmission and incidence.

## Introduction

Tick-borne viral and bacterial pathogens are major threats to human and animal health worldwide (Jongejan and Uilenberg, 2004). In Europe, changes in climate, population density, leisure activities, and agricultural practices are increasing the threat from tick-borne diseases (Gray et al., 2009, Jaenson et al., 2009, Danielova et al., 2010, Godfrey and Randolph, 2011). Understanding the interactions between pathogen and vector, and transmission from arthropod to vertebrate, may lead to novel interventions to prevent these diseases.

*Ixodes ricinus*, commonly known as the sheep tick or castor bean tick, feeds on a wide range of warm-blooded vertebrate hosts and transmits the flavivirus tick-borne encephalitis virus (TBEV), the spirochaete *Borrelia burgdorferi* sensu lato (s.l.), the obligate intracellular rickettsiae *Anaplasma phagocytophilum*, *Candidatus* Neoehrlichia mikurensis, and *Rickettsia helvetica*, and the protozoa *Babesia divergens* and *Babesia microti*. Of these zoonotic pathogens, TBEV and *B. burgdorferi* s.l. are increasingly recognised as causing serious disease in significant numbers of human patients in areas of high *I. ricinus* prevalence (Fulop and Poggensee, 2008, Sumilo et al., 2007, Czupryna et al., 2011). Surveys in Central and Eastern Europe have shown that individual ticks may be simultaneously infected with more than one of these pathogens (Alekseev et al., 2001, Reye et al., 2010, Tomanovic et al., 2010, Gern et al., 2010), but nothing is known about the effect of coinfection on pathogen replication or infectivity at the cellular level. In the closely related tick species *Ixodes persulcatus*, Alekseev et al. (2003) found a high incidence of multiple pathogen infections and suggested that *B. microti* can only survive in these ticks in the presence of coinfecting *Borrelia* spp. Popov et al. (2007) detected over 40% of unfed adult *I. persulcatus* ticks coinfected with multiple pathogens by PCR; transmission electron microscopic examination revealed cytopathic changes in salivary gland cells infected with *Ehrlichia muris* or a flavivirus, although coinfection of the same cell or organ was not observed.

Even individually, little is known about the interactions of pathogenic bacteria and viruses with ticks. Manipulation of the tick midgut and salivary gland environments in vivo by *B. burgdorferi* (Hovius et al., 2007, Schuijt et al., 2008) and *A. phagocytophilum* (Pedra et al., 2010, Sukumaran et al., 2006, Sultana et al., 2010, Ayllon et al., 2013), and of tick cells in vitro by *A. phagocytophilum* (Pedra et al., 2010, Sultana et al., 2010, Ayllon et al., 2013) have been reported. Presence of tick cells affects in vitro expression of *B. burgdorferi* s.s. outer surface proteins (Obonyo et al., 1999) and genes associated with the starvation-associated stringent response, which is usually triggered by nutritional stress such as amino acid starvation (Bugrysheva et al., 2002). Infection of *Ixodes scapularis* cell lines with the intracellular bacteria *A. phagocytophilum* or *Anaplasma marginale* causes changes in transcription levels of some host cell genes (de la Fuente et al., 2007, de la Fuente et al., 2008, Zivkovic et al., 2009, Villar et al., 2010, Ayllon et al., 2013). It has also been shown that *A. phagocytophilum* coopts ubiquitin during infection in ticks and ISE6 cells (Huang et al., 2012), which may influence cell cycle, cell viability, or replication of a second intracellular pathogen.

Much less is known about the interaction between arboviruses and tick cells in vitro. Arboviruses normally cause a persistent, low-level infection of long duration in tick cells, which is in contrast to their rapid induction of a cytopathic effect in most mammalian cells (Pudney, 1987). The maturation process of TBEV in a tick cell line was found to differ from that seen in a mammalian cell line (Senigl et al., 2006). In a recent ultrastructural study of infection of tick cells with the closely-related flavivirus Langat virus (LGTV), round vesicles and tubular structures of unknown function were associated with endoplasmic reticulum in, respectively, acute and persistent infection (Offerdahl et al., 2012).

The aim of this preliminary study was to analyse the kinetics of pathogen replication in a model system, namely tick cell cultures infected with an extracellular bacterium followed by an intracellular bacterium or a virus, and vice versa. We used a strain of the extracellular bacterium *B. burgdorferi* sensu stricto (s.s.), KS20, that is transformed with a plasmid encoding green fluorescent protein (GFP) under a highly expressed promoter (Babb et al., 2004), enabling us to easily visualise the presence of spirochaetes by fluorescence microscopy in live tick cell cultures. We used the obligately intracellular tick-borne bacterium *Ehrlichia ruminantium*, causative agent of heartwater disease in ruminants, which grows in a wide range of cell lines from different ixodid tick species (Bell-Sakyi, 2004) and can be easily visualised in Giemsa-stained cytocentrifuge smears prepared from infected cell cultures. We chose to use the mosquito-borne arbovirus Semliki Forest virus (SFV) (Togaviridae; Alphavirus) because it has been shown previously to replicate well in tick cell lines over a short 1–3-day timescale (Pudney et al., 1979, Garcia et al., 2005, Barry et al., 2013) and because there are a number of useful virus constructs available containing reporter genes such as eGFP and *Renilla* luciferase (*RLuc*) (Fragkoudis et al., 2007, Kiiver et al., 2008) allowing quick and simple monitoring of virus replication. Here, we present data on pathogen replication (relative increase or decrease) in tick cell lines derived from the *B. burgdorferi* vector species *I. scapularis* and *I. ricinus* infected sequentially with 2 of the 3 pathogens *B. burgdorferi* s.s., *E. ruminantium*, and SFV.

## Materials and methods

### Tick cell lines

All culture media and supplements were obtained from Sigma Aldrich unless otherwise indicated. The *I. scapularis* embryo-derived cell line ISE6 (Kurtti et al., 1996) was maintained at 32 °C in L-15B300 medium (Munderloh et al., 1999) supplemented with 10% tryptose phosphate broth (TPB), 5% foetal calf serum (FCS, Invitrogen), 2 mM l-glutamine (l-glut), and 0.1% bovine lipoprotein concentrate (MP Biomedicals). The *I. ricinus* embryo-derived cell line IRE/CTVM19 (Bell-Sakyi et al., 2007, Pedra et al., 2010) was maintained at 28 °C in L-15 (Leibovitz) medium supplemented with 10% TPB, 20% FCS, and l-glut. Culture media were supplemented with 100 units/ml penicillin and 100 μg/ml streptomycin except in experiments involving *B. burgdorferi*. Both cell lines were grown in 2-ml volumes in flat-sided cell culture tubes (Nunc). When required for experiments, cells of either tick cell line were seeded into wells of 24-well plates (Nunc) in 1-ml volumes of appropriate complete culture medium at a density of 6–10 × 10^5^ cells per ml and incubated overnight to allow the cells to attach. All experiments were carried out twice, with 3 or 6 replicate wells per treatment; sample time points were chosen to precede earliest expected detectable increase in *B. burgdorferi* numbers (wild-type population doubling time of 12 h, Kurtti et al., 1988) and to include expected peak [24–48 h post infection (p.i.)] and subsequent decrease of SFV replication (Pudney et al., 1979) (Table 1).

**Table 1:** Experimental design. Tick cell lines ISE6 and IRE/CTVM19 were inoculated sequentially with 2 of the arthropod-borne pathogens *B. burgdorferi* s.s., *E. ruminantium*, and Semliki Forest virus (SFV) and sampled for quantification of pathogen replication at 3–5 time points as shown. Each experiment was carried out twice, with 3 or 6 replicate wells for each treatment.

Tick cell line	First pathogen (time of inoculation)	Second pathogen (time of inoculation)	Sample time points after addition of second pathogen
ISE6	*B. burgdorferi* (0 h)	*E. ruminantium* (24 h)	24, 48, 72 h
ISE6	*E. ruminantium* (0 h)	*B. burgdorferi* (168 h)	24, 48, 72 h
ISE6	*B. burgdorferi* (0 h)	SFV (24 h)	12, 24, 48, 54, 72 h
IRE/CTVM19	*B. burgdorferi* (0 h)	SFV (24 h)	12, 24, 48, 54, 72 h
ISE6	SFV (0 h)	*B. burgdorferi* (48 h)	0, 6, 24 h
IRE/CTVM19	SFV (0 h)	*B. burgdorferi* (48 h)	0, 6, 24 h
ISE6	*E. ruminantium* (0 h)	SFV (168 h)	24, 48, 72 h
ISE6	SFV (0 h)	*E. ruminantium* (48 h)	24, 48, 72 h

### Borrelia burgdorferi s.s. infection

The *B. burgdorferi* s.s. strain KS20 (Babb et al., 2004) was grown in modified Barbour-Stoenner-Kelly medium (BSK-H) supplemented with 6% rabbit serum and 200 μg/ml kanamycin (Invitrogen) at 34 °C, with twice-weekly subculture at a dilution of 1 in 5. Prior to use in experiments with *E. ruminantium*, the spirochaetes were maintained through 2 subcultures without kanamycin as *E. ruminantium* was found to be sensitive to this antibiotic in vitro as previously reported (Van Amstel and Oberem, 1987). Tick cell culture inoculum was prepared by centrifuging the suspension of spirochaetes in complete BSK-H medium at 200 × *g* for 5 min to pellet large clumps of bacteria; 100 μl of the resultant supernatant was inoculated into each test well of the 24-well plate. Control cultures were mock-infected with 100 μl of complete BSK-H medium (without kanamycin for *E. ruminantium*-infected cells) alone.

### *Ehrlichia ruminantium* infection

*E. ruminantium* (Ball3 strain) was grown in ISE6 cells at 32 °C in flat-sided tubes as described previously for growth in IDE8 cells (Bell-Sakyi et al., 2000). In order to obtain cultures with synchronised bacterial growth, *E. ruminantium* was semi-purified from tick cells as described previously (Postigo, 2006). Briefly, infected ISE6 cells from 2 to 3 tubes were harvested by pipetting and centrifuged at 200 × *g* for 5 min at room temperature. The cell pellet was resuspended in 1 ml of 500 μg/ml trypsin in PBS and incubated for 20 min at 37 °C. Complete L-15B300 medium was added to restore the original volume and, using a syringe, the cell suspension was passed 10 times through a bent 26-gauge needle. After centrifugation at 1500 × *g* for 5 min, the supernatant was collected, and 1 ml was inoculated into each of several tubes of fresh ISE6 cells seeded on the previous day. The newly-infected ISE6 cells were incubated for 7 days before being used in experiments to ensure a sufficient infection rate (at least 5%) and relatively synchronised bacterial development. Giemsa-stained cytocentrifuge smears were prepared to confirm the presence of bacteria and absence of intact tick cells after semi-purification, and to monitor growth of *E. ruminantium* in the ISE6 cells. On day 7 p.i., at which point between 5% and 10% of cells were visibly infected, *E. ruminantium*-infected ISE6 cultures were harvested, and cells were either seeded into 24-well plates as above, or used to prepare fresh semi-purified bacterial suspension as above for inoculation (100 μl/well) into test ISE6 cultures. Control cultures were mock-infected with 100 μl/well of complete L-15B300 medium.

### Semliki Forest virus (SFV) infection

Two strains of SFV were used, one expressing *Renilla* luciferase, SFV4(3H)-*RLuc* (Kiiver et al., 2008), and the other strain expressing enhanced GFP, SFV4-steGFP (Fragkoudis et al., 2007). Virus stocks were prepared as previously described (Liljeström et al., 1991). Tick cells were infected with SFV4(3H)-*Rluc* at a multiplicity of infection of 5 by adding virus diluted in 50 μl PBS containing 0.75% albumin (PBSA) directly to the culture medium. Control cultures were mock-infected with PBSA alone. To visualise virus infection in live cells microscopically, tick cells were infected as above with SFV4-steGFP and examined 24–48 h p.i. using an Axio Observer inverted microscope (Zeiss); photomicrographs were prepared using Axiovision software (Zeiss).

### DNA extraction and quantitative real-time PCR

The entire contents (cells and supernatant medium) of each well of the 24-well plates were harvested, and DNA was extracted from 100 μl of the resultant cell suspension using a DNeasy MiniKit (Qiagen).

Quantification of *B. burgdorferi* in DNA extracted from infected tick cell cultures and control cultures without tick cells was carried out by quantitative real time PCR (qPCR). A fragment of the *B. burgdorferi* flagellin gene was amplified using the primer sequences TCTTTTCTCTGGTGAGGGAGCT (forward) and TCCTTCCTGTTGAACACCCTCT (reverse) (Pahl et al., 1999). The reaction mix contained 10 μl of Roche FastStart SYBR green master mix, 0.2 μl each of forward and reverse primers (0.4 μM), 2 μl of sample DNA, and 7.6 μl of RNAse-free H_2_O. Amplification and detection were performed with a RotorGene 3000 real-time PCR machine (Corbett Research) using the following cycle profile: 95 °C for 5 min and 45 cycles of 95 °C for 30 s, 60 °C for 30 s, 72 °C for 30 s, and a final holding step of 94 °C for 20 s. Quantification was performed by determining the threshold cycle (Ct).

Quantification of *E. ruminantium* in DNA extracted from infected and uninfected tick cell cultures was carried out by qPCR. A fragment of the 16S rRNA gene encoding for the small ribosomal subunit of *E. ruminantium* was amplified using the primers GGCAATGATCTATAGCTGGT (forward) and CTATAGGTACCGTCATTATC (reverse). The same qPCR conditions as described above were used except that the annealing temperature was decreased to 50 °C.

### Luciferase assay

Luciferase activity was measured as a marker for virus replication by dual-luciferase assay as previously described (Barry et al., 2013), using aliquots of the harvested cell suspension from each well of the 24-well plates in experiments involving SFV.

### Statistical analysis

Data were analysed across entire experiments (all time points) using Analysis of Variance (ANOVA); individual time points were analysed using Student's *t*-test. A *p* value of <0.05 was considered to be statistically significant.

## Results

**Fig. 1:**
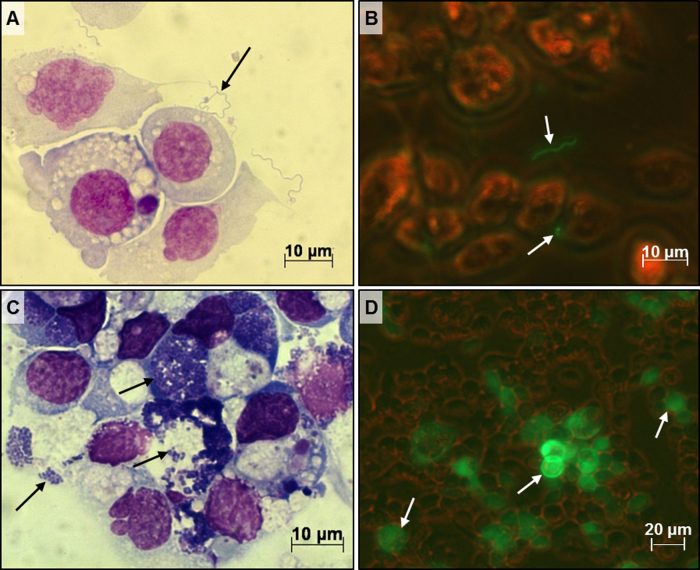
ISE6 cell cultures inoculated with *B. burgdorferi* s.s. (KS20), *E. ruminantium* (Ball 3), or Semliki Forest virus (SFV4-steGFP). (A) Giemsa-stained cytocentrifuge smear of ISE6 cells with associated *B. burgdorferi* (KS20) spirochaetes (arrow) at 24 h p.i.; (B) live ISE6 cells with *B. burgdorferi* (KS20) spirochaetes (arrows) at 6 h p.i. viewed with simultaneous brightfield and UV light; (C) Giemsa-stained cytocentrifuge smear of ISE6 cells infected with *E. ruminantium* (Ball3) (arrows) at 168 h p.i.; (D) live ISE6 cells infected with SFV4-steGFP at 24 h p.i. viewed with simultaneous brightfield and UV light; arrows indicate cells producing eGFP as a result of virus infection.

We first examined the response of tick cells to each pathogen separately by light microscopy. Tick cell cultures inoculated with *B. burgdorferi* did not show any visible adverse response (no loss of surface membrane extensions, rounding up, detachment, or lysis). Within 6 h of inoculation, most spirochaetes became associated with the surface of tick cells and remained associated throughout the experimental period (Fig. 1A). In cultures examined live by fluorescence microscopy (Fig. 1B), motile spirochaetes could easily be detected, confirming viability. To assess the potential longevity of *B. burgdorferi* in ISE6 cells, a single culture was inoculated with spirochaetes and maintained in complete L-15B300 with 10% complete BSK-H medium and weekly medium changes for 2 months at 34 °C (data not shown). Motile, cell-associated spirochaetes were observed by fluorescence microscopy up to day 60, and the ISE6 cells remained healthy-looking throughout. Real-time qPCR analysis revealed that there was no detectable multiplication of *B. burgdorferi* in any of the cultures with tick cells either singly or coinfected during the maximum 4-day experimental period, while control cultures of spirochaetes alone in 100% BSK-H medium had multiplied on average 4-fold by 72 h p.i. (data not shown). Growth of *E. ruminantium* in ISE6 cells was similar to that described previously in another *I. scapularis* cell line (IDE8) (Bell-Sakyi et al., 2000) and in cell lines from 5 other tick species (Bell-Sakyi, 2004). No cytopathic effect was seen by inverted microscope examination in live tick cells infected with *E. ruminantium* (Fig. 1C) or SFV (Fig. 1D) throughout the experimental periods of 10 and 3 days, respectively.

### Infection with *B. burgdorferi* s.s. followed by *E. ruminantium* and vice versa

**Fig. 2:**
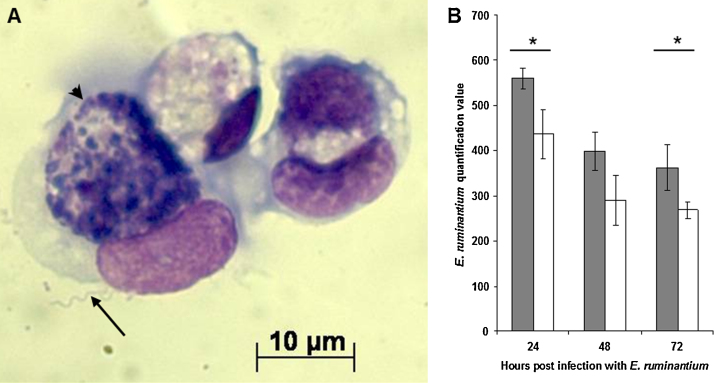
Infection of ISE6 cells with *B. burgdorferi* s.s. and *E. ruminantium*. (A) *B. burgdorferi* spirochaete (arrow) “interacting” with an ISE6 cell containing a colony of *E. ruminantium* (arrowhead). (B) Quantification of *E. ruminantium* in ISE6 cells infected with *B. burgdorferi* followed 24 h later by *E. ruminantium*. Shaded bars: cells infected with *B. burgdorferi* and *E. ruminantium*; white bars: cells infected with *E. ruminantium* alone. The amount of *E. ruminantium* DNA detected by real-time PCR was significantly higher in cells with *B. burgdorferi* than in cells with *E. ruminantium* alone at 24 and 72 h p.i. (Student's *t*-test) and across all time points (ANOVA, *p* = 0.0012). Experiment was repeated twice with similar results; 3 replicates per treatment, error bars represent standard deviation. * indicates *p* value <0.05 by Student's *t*-test.

In ISE6 cell cultures inoculated or mock-inoculated with *B. burgdorferi* and 24 h later inoculated with semi-purified *E. ruminantium*, there was a decrease in the amount of *E. ruminantium* detected by real-time qPCR 24 h later in all cultures (Fig. 2). However, there was a significant difference between the 2 groups at 24 and 72 h p.i. (Student's *t*-test). ANOVA indicated that amounts of *E. ruminantium* were significantly higher across all time points (24, 48, and 72 h p.i.) in the presence of *B. burgdorferi* (*p* = 0.0012) compared to cultures with *E. ruminantium* alone. In ISE6 cultures infected first with semi-purified *E. ruminantium*, addition of *B. burgdorferi* 7 days later had no effect on the amount of *E. ruminantium* detected over the subsequent 72 h (data not shown). Thus, the decrease in *E. ruminantium* replication was less in cultures already infected with *B. burgdorferi* than when *B. burgdorferi* was subsequently added to *E. ruminantium*-infected cells.

### Infection with *B. burgdorferi* s.s. followed by SFV and vice versa

**Fig. 3:**
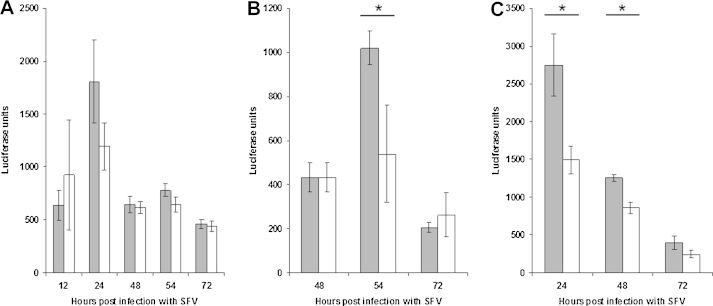
*Renilla* luciferase activity in tick cells infected with a bacterium and SFV4(3H)-*Rluc* (Semliki Forest virus). Shaded bars: cells infected with a bacterium and SFV; white bars: cells infected with SFV alone. Experiments were repeated twice with similar results; 3 replicates per treatment, error bars represent standard deviation. (A) IRE/CTVM19 cells infected with *B. burgdorferi* s.s. followed 24 h later by SFV4(3H)-*Rluc*. Luciferase activity peaked in all cultures at 24 h p.i., and there was an increase in activity of cells with *B. burgdorferi*, but it was not significant (Student's *t*-test, *p* = 0.07). The difference in activity between cells with and without *B. burgdorferi* was not significant over all time points (ANOVA). (B) *Renilla* luciferase activity in IRE/CTVM19 cells infected with SFV4(3H)-*Rluc* followed 48 h later by *B. burgdorferi*. Luciferase activity was significantly higher 6 h after addition of *B. burgdorferi* to SFV-infected cells compared to cells with SFV alone (Student's *t*-test, *p* < 0.05), while by ANOVA there was no significant difference across all time points. (C) *Renilla* luciferase activity in ISE6 cells infected with *E. ruminantium* followed 7 days later by SFV4(3H)-*Rluc*. Luciferase activity was highest in all cultures at 24 h p.i. with significantly higher activity in cells coinfected with *E. ruminantium* and SFV compared to cells infected with SFV alone at 24 and 48 h p.i. (Student's *t*-test) and across all time points (ANOVA, *p* = 0.0028). * indicates a *p* value <0.05 by Student's *t*-test.

Both cell lines, ISE6 and IRE/CTVM19, were inoculated or mock-inoculated first with *B. burgdorferi* and then 24 h later with SFV4(3H)-*RLuc*. Luciferase activity was measured at 12, 24, 48, 54, and 72 h p.i. with SFV. In both cell lines, the highest level of luciferase activity (an indicator of virus activity) was seen at 24 h p.i. However, there was no significant difference in luciferase activity between cultures with and without prior *B. burgdorferi* infection. Fig. 3A shows the results for IRE/CTVM19. When both cell lines were infected first with SFV4(3H)-*Rluc* and then inoculated at 48 h p.i. with *B. burgdorferi*, a peak in luciferase activity was seen 6 h later (54 h p.i. with SFV). In IRE/CTVM19 cells (Fig. 3B), this peak of luciferase activity was significantly higher (Student's *t*-test, *p* < 0.05) in cultures that had been subsequently infected with *B. burgdorferi*, compared to mock-infected cultures. Overall, addition of *B. burgdorferi* to virus-infected cells tended to result in increased virus activity in both cell lines.

### Infection with *E. ruminantium* followed by SFV and vice versa

ISE6 cells were first inoculated with semi-purified *E. ruminantium* and 7 days later with SFV4(3H)-*Rluc*. There was no significant difference between virus-infected and mock-infected cultures in the amount of *E. ruminantium* detected by real-time qPCR after a further 24 h, 48 h, or 72 h, although in both cases there was a decline in the amount of *E. ruminantium* over time (data not shown), as seen in cultures inoculated first with *B. burgdorferi* and then with *E. ruminantium*. However, replication of SFV as measured by *Renilla* luciferase activity was significantly higher at 24 and 48 h p.i. (Student's *t*-test) and across all time points (ANOVA, *p* = 0.0028) in cells with prior *E. ruminantium* infection compared to cultures infected with SFV alone (Fig. 3C). In contrast, inoculation of ISE6 cells with semi-purified *E. ruminantium* 48 h after SFV infection had no effect on replication of SFV nor on the amount of *E. ruminantium* detected over the subsequent 72 h (data not shown).

## Discussion

This preliminary study examined whether bacterial and viral pathogens coinfecting tick cells in vitro would interact to favour replication of one or both microorganisms, a possibility which, if also occurring in vivo, could result in increased likelihood or rate of transmission of the favoured pathogen. Of the 6 combinations tested, we found 3 in which presence of one pathogen significantly enhanced replication of the other. When *E. ruminantium* was added to tick cell cultures previously inoculated with *B. burgdorferi* s.s., the amount of *E. ruminantium* DNA was consistently greater over the following 3 days compared to cultures without spirochaetes. Similarly, SFV replication in tick cells was enhanced following addition of extracellular *B. burgdorferi* or when the cells were already infected with intracellular *E. ruminantium* at the time of virus infection, but not vice versa. Further study will be needed to determine whether the observed enhancement of replication is due to direct pathogen–pathogen interaction, or to indirect effects on the host cell innate immune response, or a combination of these.

Of the pathogens used in the present study, only *B. burgdorferi* is transmitted by *Ixodes* ticks. Although the other 2 pathogens are not naturally associated with ticks of this genus, we chose to use them as model systems for the following reasons. *Ehrlichia ruminantium* is an obligately intracellular bacterium in the family Anaplasmataceae, closely related to *A. phagocytophilum* (Dumler et al., 2001); it is transmitted by ixodid ticks of the genus *Amblyomma* and, like *A. phagocytophilum*, infects neutrophils in the mammalian host and can be propagated in tick cell lines (Bell-Sakyi, 2004, Munderloh et al., 1999). In vitro culture of *E. ruminantium* in many tick cell lines, including those from *I. scapularis* and *I. ricinus*, is well established in our laboratory (Bell-Sakyi, 2004), and the parameters relating to infection and growth rate in tick cells are defined. Moreover, knowledge of the bovine pathogen *A. marginale* has been greatly expanded through extensive in vitro studies carried out in *I scapularis* cell lines, even though this tick species is not a natural vector (Blouin et al., 2002, Bell-Sakyi et al., 2007). Recently, another biologically irrelevant model system, infection of the *Drosophila* S2 cell line with the tick-borne human pathogen *Ehrlichia chaffeensis*, has yielded information about differential transcription of arthropod host cell genes (Von Ohlen et al., 2012). Ideally, our experiments should be repeated using, for example, the *Ixodes*-transmitted *A. phagocytophilum* as the intracellular bacterium (Munderloh et al., 1996, Munderloh et al., 1999, Woldehiwet et al., 2002); however, we did not have access to in vitro cultures of this pathogen in tick cells at the time our study was carried out. The virus used, mosquito-borne SFV, is not known to be tick-transmitted, but replicates in a range of ixodid tick primary cultures and cell lines (Bell-Sakyi et al., 2012, Pudney et al., 1979) including the lines used in this study (Barry et al., 2013). Furthermore, SFV has recently been isolated from 4 ixodid tick species collected from mammalian hosts in Kenya (Lwande et al., 2013), and experimental transmission of the closely-related Venezuelan equine encephalitis virus (Togaviridae; Alphavirus) to guinea pigs by ixodid ticks has been reported (Linthicum et al., 1991, Linthicum and Logan, 1994). The particular advantage of SFV was the availability in our laboratory of genetically modified constructs of this virus incorporating various reporter genes such as those encoding eGFP and luciferase (Fragkoudis et al., 2007, Kiiver et al., 2008), which allowed us to assay viral parameters in the tick cell cultures much more quickly and easily than by traditional plaque assay or immunostaining methods. The system we describe here may be useful for further studies with more biologically relevant pathogens such as the tick-borne virus TBEV or its less pathogenic relative LGTV in combination with *A. phagocytophilum* and/or *B. burgdorferi* s.l.

When cocultivated with tick cell lines, the population doubling time of wild-type *B. burgdorferi* was around 2.5 times slower (27.1 h) than axenic growth in BSK-H medium (11.7 h) (Kurtti et al., 1988). We used a strain of *B. burgdorferi* s.s., KS20, transformed with a plasmid-expressing GFP (Babb et al., 2004) because it facilitated rapid confirmation of presence and location of spirochaetes in live tick cell cultures. We did not detect appreciable *B. burgdorferi* multiplication over 72 h of cocultivation with tick cells, while spirochaetes cultured axenically in 24-well plates with BSK-H medium increased 4-fold in the same time period as measured by PCR; this growth rate is much slower than that of wild-type spirochaetes, which would be expected to increase approximately 32-fold in 72 h (Kurtti et al., 1988). Even allowing for the adverse effects of cultivation in an unsealed 24-well plate compared to a sealed tube with a high ratio of medium to airspace, this suggests that the transformation affected the fitness of the KS20 spirochaetes. In previous studies, wild-type *B. burgdorferi* induced a pronounced cytopathic effect in tick cell lines, including ISE6, grown at 34 °C, with rounding up and detachment of cells within 5–7 days (Obonyo et al., 1999, Kurtti et al., 1993). In contrast, we did not observe any appreciable cytopathic effect in ISE6 or IRE/CTVM19 cells during cocultivation with KS20 strain *B. burgdorferi* s.s. for up to 2 months. It is possible that growth of the KS20 spirochaetes and concomitant cytopathic effect on tick cells could have been enhanced by supplementation of the L-15 and L-15B300 media with 2.23 mM N-acetylglucosamine as used by Kurtti et al. (1988).

The effect of infection with an intracellular bacterial tick endosymbiont on host cell interaction with *B. burgdorferi* was studied in 3 tick cell lines by Mattila et al. (2007). Two *I. scapularis* cell lines, IDE12 and ISE6, were found to respond very differently to the spirochaetes, with IDE12 cells phagocytosing a much higher proportion of bacteria than ISE6 cells. Infection with the endosymbiont *Rickettsia peacockii* had little or no effect on survival or phagocytosis of the spirochaetes either in IDE12 cells (derived from a natural vector of *B. burgdorferi*) or in the cell line DAE15 (derived from the natural host of *R. peacockii*, the tick *Dermacentor andersoni*). This supports our finding that in vitro growth of *B. burgdorferi* s.s. did not differ between uninfected tick cell cultures and cultures infected with the intracellular bacterial pathogen *E. ruminantium*. Effect of spirochaetes on *R. peacockii* growth was not assessed (Mattila et al., 2007).

In summary, this preliminary study using a model system revealed that the presence of extracellular *B. burgdorferi* s.s. spirochaetes in tick cell cultures enhanced short-term replication in vitro of 2 intracellular pathogens, *E. ruminantium* and SFV, while the presence of the intracellular pathogens had no effect on extracellular spirochaete replication. SFV also replicated to higher levels in the presence of the intracellular bacterium *E. ruminantium*, but the presence of SFV had no effect on bacterial replication. Further experiments in which the tick cell responses to the various microorganisms are characterised would help to determine whether or not there is true synergistic interaction between the investigated pathogens, and could lead to novel approaches to control of ticks and tick-borne diseases similar to that involving *Wolbachia* and dengue virus in mosquitoes (Iturbe-Ormaetxe et al., 2011). Furthermore, study of experimental coinfections of *Ixodes* ticks with different combinations of bacterial and viral pathogens of biological relevance is required to confirm whether observations in vitro can be extrapolated to the in vivo situation, and to what extent processes such as saliva-activated transmission of pathogens (Nuttall and Labuda, 2004) may be influenced by coinfections.
